# Usability evaluation of mobile applications using ISO 9241 and ISO 25062 standards

**DOI:** 10.1186/s40064-016-2171-z

**Published:** 2016-04-29

**Authors:** Karima Moumane, Ali Idri, Alain Abran

**Affiliations:** Software Project Management Research Team, ENSIAS, University Mohammed V, Rabat, Morocco; Department of Software Engineering and IT, Ecole de Technologie Supérieure, Montreal, H3C IK3 Canada

**Keywords:** ISO 25062, ISO 9241, Mobile usability evaluation, Software measures

## Abstract

This paper presents an empirical study based on a set of measures to evaluate the usability of mobile applications running on different mobile operating systems, including Android, iOS and Symbian. The aim is to evaluate empirically a framework that we have developed on the use of the Software Quality Standard ISO 9126 in mobile environments, especially the usability characteristic. To do that, 32 users had participated in the experiment and we have used ISO 25062 and ISO 9241 standards for objective measures by working with two widely used mobile applications: Google Apps and Google Maps. The QUIS 7.0 questionnaire have been used to collect measures assessing the users’ level of satisfaction when using these two mobile applications. By analyzing the results we highlighted a set of mobile usability issues that are related to the hardware as well as to the software and that need to be taken into account by designers and developers in order to improve the usability of mobile applications.

## Background

In 2012, the average use of smart phones increased by 81 % over 2011 and the average download of mobile applications increased to 342 MB per month and per smartphone, compared to 189 MB in 2011 (Cisco Visual Networking Index 2014) at an international level. The Gartner group study (2014) reports a 2011 yearly 19 % rate of increase in sales of mobile devices. Furthermore, in 2013 nearly 102 billion of mobile applications were downloaded, versus 64 billion in 2012. In 2017, it is expected that this number will increase to 254 billion download (Gartner 2014). These statistics show that smart phones and tablets have invaded the daily lives of consumers: at home, at work, and in public places. Indeed, according to the data provided by Sales Force Marketing Cloud (2014), 85 % of people with smart phones consider their devices an inseparable part of their lives.

Consequently, the growing number of mobile users automatically influences the growth of mobile applications (i.e., apps) that are available in the download platforms, such as the App Store and the Play Store.

In our earlier framework on the use of the software quality standard ISO 9126 in mobile environments, we had identified several mobile limitations that may affect the quality of apps, some of which often have a negative effect on the usability of apps such as smaller screen size, low display resolution, the context in which the mobile device is used and low memory (Idri et al. 2013). Therefore, the evaluation of usability of apps, which must be initiated before the launch of the apps, is considered as a new area of research, (Kjeldskov and Stage 2004). Usability evaluation and remedial actions can help developers to meet the needs of users by designing easy to use apps.

However, few studies have been carried out on the use of ISO 25062 and ISO 9241 to evaluate the software quality of apps and to address the limitations of mobile environments. Most have focused on evaluating the usability of very specific types of apps, such as the Satnav applications (Hussain and Kutar 2009, 2012a), mobile geo-applications (van Elzakker et al. 2008), and mobile tourism applications (Ahmadi and Kong 2008; Geven et al. 2006; Schmiedl et al. 2009; Shrestha 2007; Echtibi et al. 2009).

The aim of this study is to present an empirical evaluation of our framework developed on the use of the software quality standard ISO 9126 in mobile environments (Idri et al. 2013), especially the influence of mobile limitations of these environments on the usability of apps running on existing operating systems (OS). To do that, we have relied on the ISO 9241-11:1998, ISO 25062:2006 standards for the usability evaluation. Thirty-two users have participated in the experiment with different types of smart phones (Android, iOS, etc.). They have been asked to perform a set of defined tasks for Google Apps and Google Maps, which were selected as test cases to investigate usability problems. The Questionnaire for User Satisfaction Interaction (QUIS 7.0) (Hussain and Kutar 2012a) was used to assess the user’s satisfaction level.

The paper is structured as follows: “Using ISO 9126 for software quality in mobile environments” section presents the challenges of mobile environments and describes our earlier framework based on ISO 9126 to determine the software quality characteristics that may be influenced by the mobile environments limitations. “Usability evaluation of mobile applications” section defines the usability evaluation of apps according to ISO standards, presents the existing usability evaluation methods, and describes the components of the context of use as proposed by ISO 9241. “Experiment design” section describes the design of the experiment. “Discussion and interpretation” section discusses the results and presents a set of mobile usability issues that must be taken into consideration by developers in the design of user-friendly mobile applications in addition to the presentation of threats to validity. Finally, findings are discussed and future works are presented in “Conclusion and future work” section.

## Using ISO 9126 for software quality in mobile environments

**Fig. 1:**
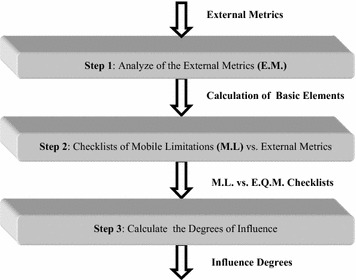
Analysis process (Idri et al. 2013)

In our previous study (Idri et al. 2013), we have developed a framework in order to use the ISO 9126, particularly its external quality model, to deal with mobile environments limitations which are mainly decomposed into two subcategories: (1) mobile devices limitations such as, limited energy autonomy, limited user interface and limited storage capacity; and (2) wireless networks limitations which are as follows: frequent disconnection, lower and variable bandwidth. The framework developed is based on an analysis process which was designed to take into consideration the limitations of mobile environments. The process is applied to the six external quality characteristics: functionality, reliability, usability, efficiency, maintainability, and portability and consists of three steps as shown in Fig. 1 (Idri et al. 2013).

 Table 1 shows the results of the application of the analysis process on the six characteristics of ISO 9126 external quality. We noted that:

**Table 1:** Evaluation of the degree of influence of the mobile limitations on ISO 9126 characteristics (Idri et al. 2013)

Characteristic	Frequent disconnection	Low bandwidth	Variable bandwidth	Limited autonomy of energy	Limited capacity of storage	Limited user interface
Functionality					X	X
Reliability	X		X	X		
Usability						X
Efficiency		X			X	
Maintainability	More external metrics should be provided, in particular for the attributes of stability and testability					
Portability					X More external metrics must be proposed

1. Reliability is influenced by the following mobile limitations: frequent disconnection, variable bandwidth, and limited energy autonomy. Therefore, during the evaluation of this characteristic, these three mobile limitations must be supported using the ISO 9126 measures or by providing other ones specific to mobile environments. The same goes for the Efficiency characteristic (lower bandwidth and limited storage capacity), and the usability characteristic (limited user interface).
2. Usability is influenced just by limited user interface, since we have observed that most of usability external metrics are influenced by limited user interface (21 of 39 metrics) (Idri et al. 2013).
3. Functionality is not impacted by the mobile limitations considering recommendations presented in ISO 25010. Therefore, this characteristic can be evaluated in the same way as in a fixed environment.
4. Portability is impacted by limited storage capacity and limited user interface. When evaluating this characteristic these two limitations must be taken into account by proposing new measures specific to mobile environments.
5. No recommendations have been made for the maintainability characteristic. ISO 9126 should provide additional measures, particularly for the stability and testability attributes to decide whether or not there is an influence relationship between maintainability and mobile limitations.

This work presents an empirical validation of our framework findings as shown in Table 1, especially those concerning the usability characteristic. The choice was made in the first place on the usability, since it is a key characteristic for an application to be learned, attractive and easy to use by final users. Therefore, we evaluate the usability of mobile applications taking into consideration the limited user interface since it is related to the usual challenges of the apps. In contrast to the study in Idri et al. (2013) that used the External Quality Model of ISO 9126 but without taking into consideration the context of use, this empirical validation also take into account other mobile limitations such as:

- *Mobile context* it concerns “any information that characterizes a situation related to the interaction between users, applications, and the surrounding environment (Dey et al. 2001)”. It includes everything that can distract users’ attention such as environment, persons, etc. It is not obvious to include all possibility of the context in one mobile usability evaluation (Longoria 2001).
- *Network characteristics* Limited and variable bandwidth of networks is a common obstacle for mobile applications (Longoria 2001) that affect the data download time, the quality of audio and video streams, in addition to the data transfer and the signal power which depends on the mobility of users (Sears and Jacko 2000).
- *Screen size* The small size of mobile devices screens has a significant impact on the usability of mobile applications (Kim and Albers 2001).
- *Display resolution* Low display resolution of mobile devices affects the display quality on the screen of multimedia data and files. Therefore, different mobile devices with different display resolution screens can produce different results of mobile usability evaluation.
- *Limited storage capacity* The memory capacity and computing power of mobile devices are incomparable to computers. Therefore applications that require high-capacity memory for installation, graphics or fast processing speed remain impractical on mobile devices (Rakkolainen and Vainio 2001).
- *Data entry techniques* Small graphics like the small buttons and labels reduces the input speed data which increases the errors and therefore limit the effectiveness and the efficiency of the user when entering data (Longoria 2001; MacKenzie and Zhang 1999; Soukoreff and MacKenzie 1995).

## Usability evaluation of mobile applications

According to ISO 9126, the usability characteristic is defined as “the capability of the software product to be understood, learned, used, and attractive for the user, when used under specified conditions” (ISO 9126-2 2001); it is subdivided into four sub-characteristics:

1. Understandability: “the capability of the software product to enable the user to understand whether the software is suitable, and how it can be used for particular tasks and conditions of use”.
2. Learnability: “the capability of the software product to enable the user to learn its application”.
3. Operability: “the capability of the software product to enable the user to operate and control it”.
4. Attractiveness: “the capability of the software product to be attractive to the user”.

ISO 25010 standard constitutes a revision of ISO 9126-1: 2001. The revised ISO software product quality model is composed of two parts: (1) the internal and external software quality model and (2) the quality in use model (ISO/IEC 25010-2 2008). It’s including the same characteristics of the software quality with some modifications: more specifically, the quality of use that has been divided into usability in use, flexibility in use and safety. The usability in use includes the effectiveness in use, the efficiency in use, the satisfaction in use and the usability in use compliance (ISO/IEC 25010-2 2008).

In 2006, the Common Industry Format (CIF) for usability was adopted by ISO as part of the ISO 25000 series. The CIF provides a set of standards, such as ISO 25060 for the specification and the evaluation of the usability of interactive systems (ISO/IEC TR 25060 2010), and the ISO 25062 as a standard method for reporting results of usability evaluation (ISO/IEC 25062 2006). The report components and format of the CIF are consistent with the definition of usability according to ISO 9241-11 standard: “the extent to which a product can be used by specified users to achieve specified goals with Effectiveness, Efficiency and Satisfaction in a specified Context of use” (ISO 1998).

- Effectiveness: “the accuracy and completeness with which specified users can achieve specified goals in particular environments”.
- Efficiency: “the resources expended in relation to the accuracy and completeness of goals achieved”.
- Satisfaction: “the comfort and acceptability of the work system to its users and other people affected by its use”.

These definitions of effectiveness, efficiency and satisfaction in ISO 9241 are similar to the definitions in ISO 9126. However, in ISO 9126, they are defined on the basis of the software product, where as in ISO 9241-11 they are based on the users’ views and opinions (ISO/IEC 25062 2006). These findings further justifies the use of ISO 9241-11 for the usability evaluation; similarly, Constantinos and Dan (2007) analyzed the dimensions of usability measures and found that the main components of usability evaluation are indeed effectiveness, efficiency, and satisfaction with percentages of 62, 33 and 20 % respectively, in addition to the context of use which is also an important component to consider when evaluating the usability.

### Context of use

In ISO 9241(ISO/IEC 25062 2006; ISO 1998), the context of use includes the following:

- *Users* There are several user characteristics that influence the use of a mobile application: (1) the experience with a particular type of device and a specific mobile application (Constantinos and Dan 2007; Mao et al. 2005) (experienced or a novice user); (2) the age; (3) the gender; (4) the level and the nature of education; and (5) the occupation (Suzuki et al. 2009; Ling et al. 2006).
- *Tasks* A set of tasks are defined to be executed by users to evaluate the usability of a mobile application.
- *Device/Equipment* A mobile device differs from another with several aspects of form and design as the keyboard type (manual or virtual), screen sizes, colors, and storage capacity. Also a mobile application can be launched on a smartphone, or on a tablet under different OS; hence, the choice of the device category and the type of OS should be considered (Ryan and Gonsalves 2005).
- *Environment* As explained below in “Usability evaluation methods and techniques” section, the evaluation of usability can be achieved in a laboratory setting or in the field that is a real world. However, all user interactions with the devices/apps are important, and must be recorded for interpretations and analysis.

### Usability evaluation methods and techniques

There are four types of methods frequently used for the usability evaluation, each with specific characteristics (Preece 1993):

1. *Heuristic evaluation or experts-based evaluation* it is a method of usability evaluation carried out by one or more human experts to describe the problems that may be encountered by inexperienced users when using an interactive system.
2. *Observation* it involves the collection of data relating to what users do when interacting with an interface by using video recording, thinking aloud protocol or direct observation.
3. *Surveys* are used to identify the users’ views and feedbacks to understand their expectations for a given product, using questionnaires and interviews.
4. *Experimental evaluation* an evaluation method that can be conducted by experts and/or users to address the mobile usability issues using questionnaires, interviews and software logging.

These methods can be applied in two different ways: laboratory tests and field tests (Kaikkonen et al. 2005). In a laboratory experiment, users must perform tasks relating to a mobile application in a very specific and controlled environment, away from interruptions, noise, etc. Hence, the control of the experiment and data collection is not an issue, but it is nonetheless unrealistic. In contrast, the field evaluation tests allow participants to really use the apps (Johnson 1998a; Tamminen et al. 2004). However, the evaluation of usability in the field is not easy (Nielsen 1998; Brewster 2002). Kjeldskov and Graham (2003) conducted a major study which showed that 71 % of usability apps evaluations were performed in laboratory settings because of the complexity of data collection in the field, as users move physically (Johnson 1998b; Petrie et al. 1998) and it is not obvious to apply pre-established Evaluation techniques, such as direct observation and video recording (Pascoe et al. 2000).

## Experiment design

The Limited User Interface is one of the key constraints of mobile devices as they are generally with small size (Oehl et al. 2007). This means that the display capacity of smart phones, for instance, is very restricted, which requires that the designer adapt the human machine interface of apps to this constraint. For evaluating the influence of this limitation on the mobile usability and in order to highlight the influence of the other mobile limitations (“Using ISO 9126 for software quality in mobile environments” section), an experiment was designed and ran using direct observation of users, video recordings in addition to questionnaires. This Empirical Evaluation was based on the ISO 9241-11:1998, ISO 25062:2006 using the experimental process proposed by Wholin et al. (2012), Kitchenham et al. (2002).

This experiment is aimed at answering two questions: (1) what are the measures that have been impacted during the evaluation of the usability of mobile applications? (2) what are the other mobile limitations that influence the usability of mobile applications compared to limited user interface? The different steps of the experiment are discussed in the following sections.

### Experiment subjects

**Fig. 2:**
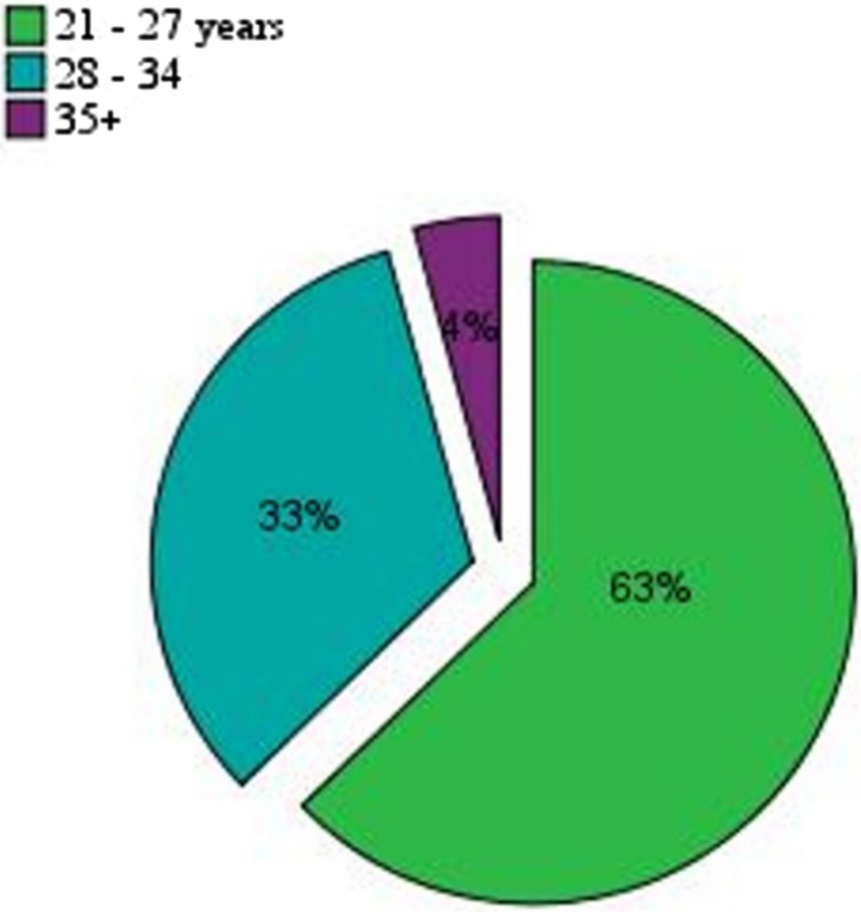
Distribution of users by age

The participation in this experiment of different users, from a beginner to an expert, enables us to have different opinions, different reactions, as well as inconsistent feedbacks when using the apps (Nielsen 1994). However, the number of users depends on several parameters such as cost, time, equipment, and effort required for the collection and analysis of data (Dumas and Redish 1999). In this study, 32 users whose age varies from 20 to 45 have participated in the experiment, including men and women, as suggested by Nielson (1994; Nielsen and Landauer 1993). Ten of them were novice, including two who did not have their own smartphones, and used those of their colleagues. A research hypothesis is that these ten users will have difficulties during the execution of tasks; therefore, they would spend more time in the execution of tasks and may make errors also. There was also a participant with disabilities: it allowed addressing the problems related to the usability of apps by people with limited capabilities. In addition, another participant had experience in apps development; therefore, he has an idea about the development of mobile interfaces. He will be considered as an expert in this study and it is assumed that he will perform tasks quickly with minimal errors. As shown in Fig. 2, 96 % of the participants were between the ages of 21 and 34, the remaining were over the age of 35.

**Fig. 3:**
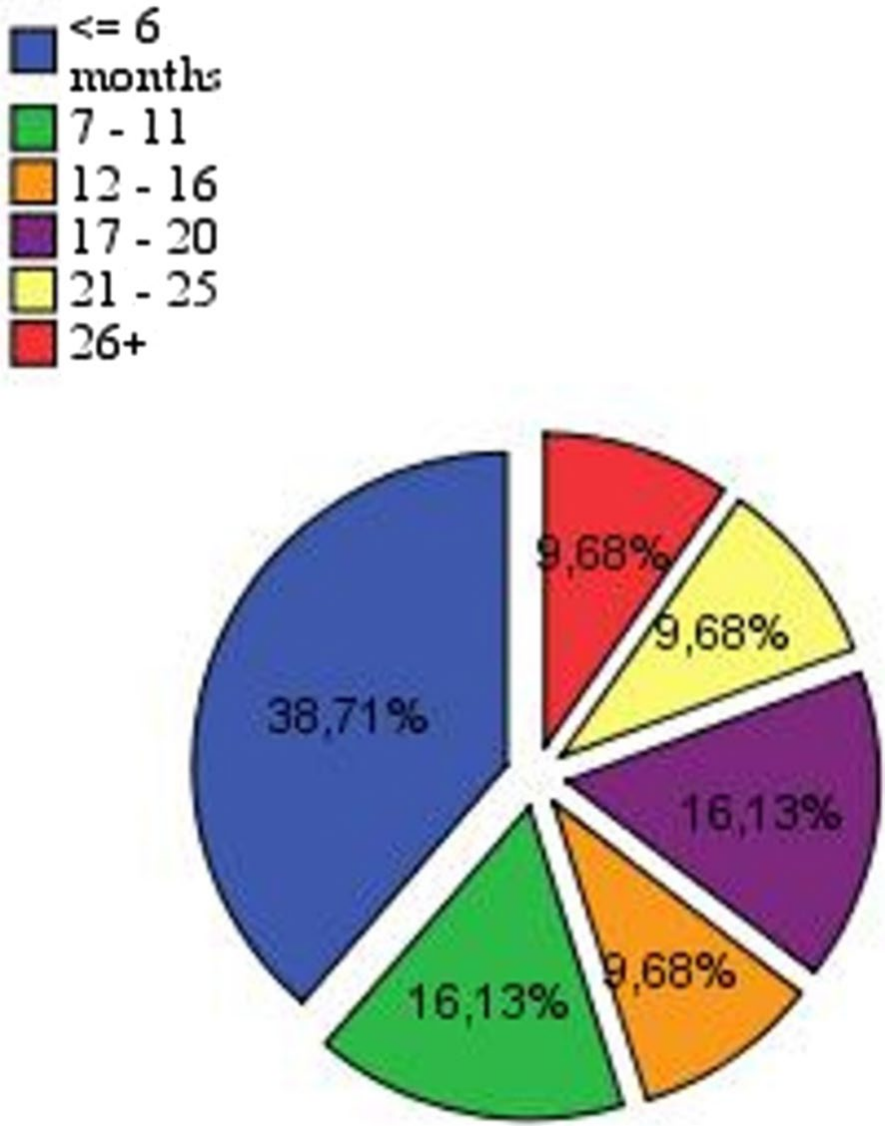
Distribution of users by the possession period of mobile devices

To determine the level of experience of participants in the use of their smart phones, the following question was asked: “how long have you possessed this smart phone?” The answers range from 15 days to 3 years. Therefore, people who own their devices for a greater length of time manipulate their devices correctly, which helps them to perform tasks more quickly and reliably. Figure 3 shows the number of participants according to the period of mobile device possession: 38.71 % of participants owned their smart phones for less than 6 months and only 9.68 % of participants have their smart phones for more than 2 years.

**Fig. 4:**
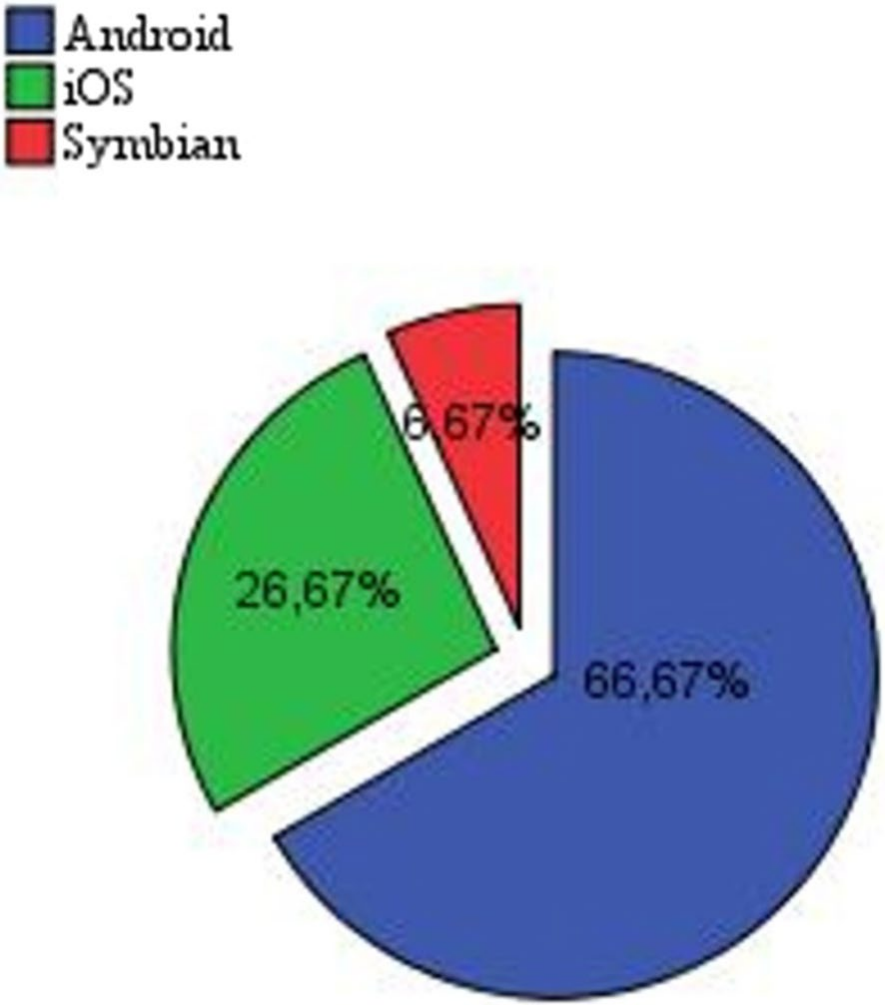
Distribution of users by types of OS devices

The aim of this study is to evaluate the influence of screen size of mobile devices on the usability of apps, and to ensure that the identified issues of usability are not related to a very specific platform. To do that, the experiment was opened to users with devices under various OS platforms. Figure 4 shows the distribution of participants by platform. The Android is the most used platform with 66.67 % of participants followed by iOS with 26.67 %. The Android devices used in this study were: S4, S3, S3 Note, S3 mini, S2, S1, S young and Htc. Regarding iOS ones, they were Iphone 5/5S and Iphone 4/4S in addition to Nokia E72 and Nokia Music as Symbian devices.

### Independent and dependent variables

The experimental evaluation was based on ISO 9241-11:1998, ISO 25062:2006. Effectiveness and efficiency will be measured and collected via video recordings of users performing tasks, in the form of the objective measures shown in Table 2. Regarding satisfaction, it is measured subjectively through questionnaires.

**Table 2:** Usability Objective and Subjective measures

Attribute	Metrics	Description
Effectiveness	Time to learn and use	Time to read the scenarios and to begin performing tasks
	Data entry time	Time to enter the data necessary for the execution of a task
	Tasks time	Time to accomplish given tasks
	Response time	Time of having the response to the requested information
	Time to install	Installation time of applications or its update
Efficiency	Number of errors	Number of errors made while reading scenarios and during the task execution
	Completion rate	The percentage of participants who correctly complete and achieve the goal of each task
Satisfaction	Questionnaires	The QUIS v 7.0 (“Instrumentation” section)

- *Dependent variables* the dependent variables in this experiment are the objective and subjective measures used for evaluating the mobile usability, see Table 2.
- *Independent variables* the groups of users and their level of experience, the different mobile devices and types of OS in addition to mobile limitations (“Using ISO 9126 for software quality in mobile environments” section) are the main independent variables in this experiment.

### Instrumentation

Two apps were used in this empirical evaluation: (1) Google Maps which was proposed by some participants: it is the default GPS installed on most smart phones and (2) Google Apps seen its popularity and diversity in all the fields.

Moreover, both apps are compatible and supported by all types of OS and have been used in several evaluation works on mobile usability (Hussain and Kutar 2012a, b). The selection of tasks for each application was based on the most frequent tasks and the most used, including most troublesome tasks that require more concentration.

**Fig. 5:**
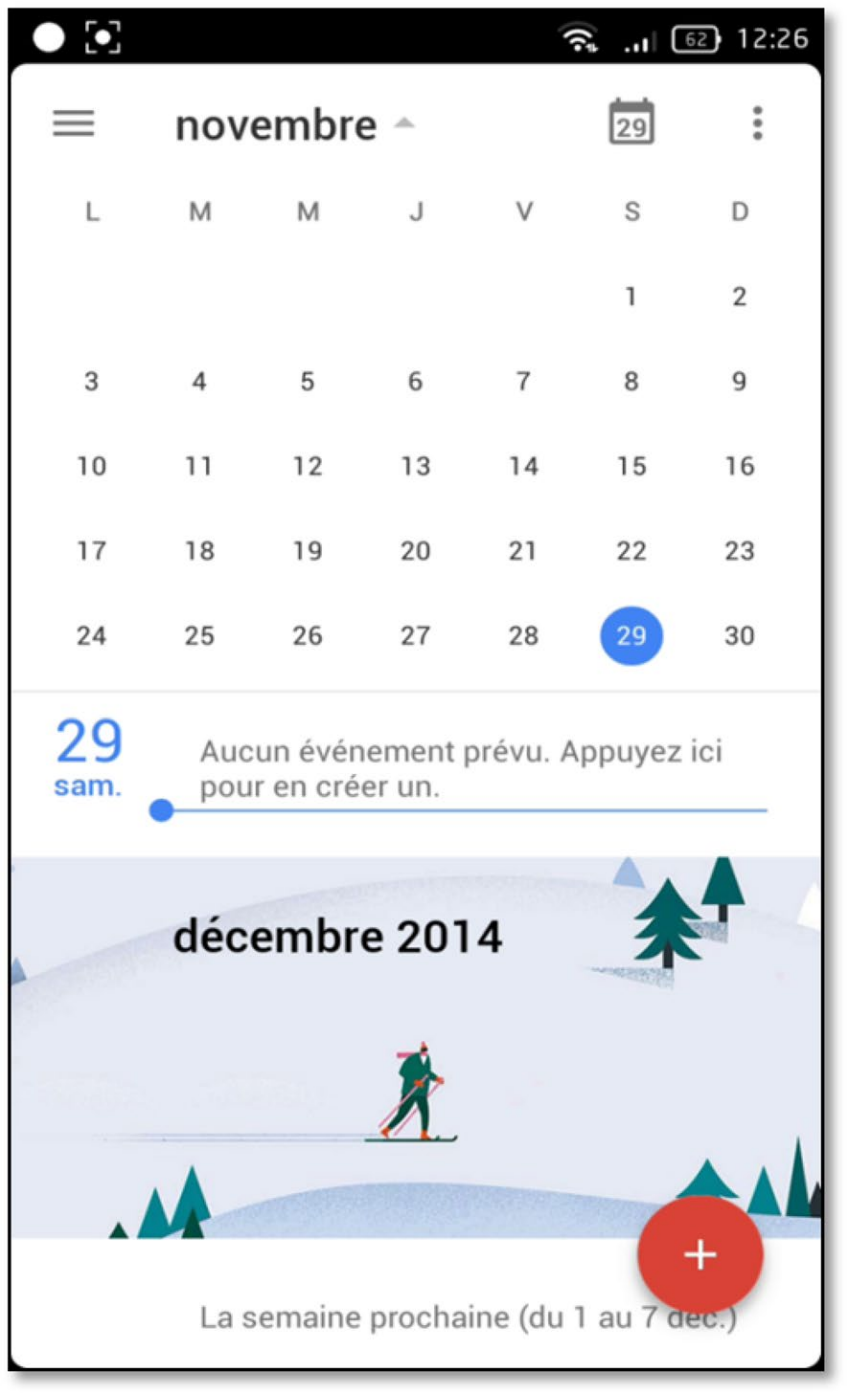
Google agenda on Android device

**Fig. 6:**
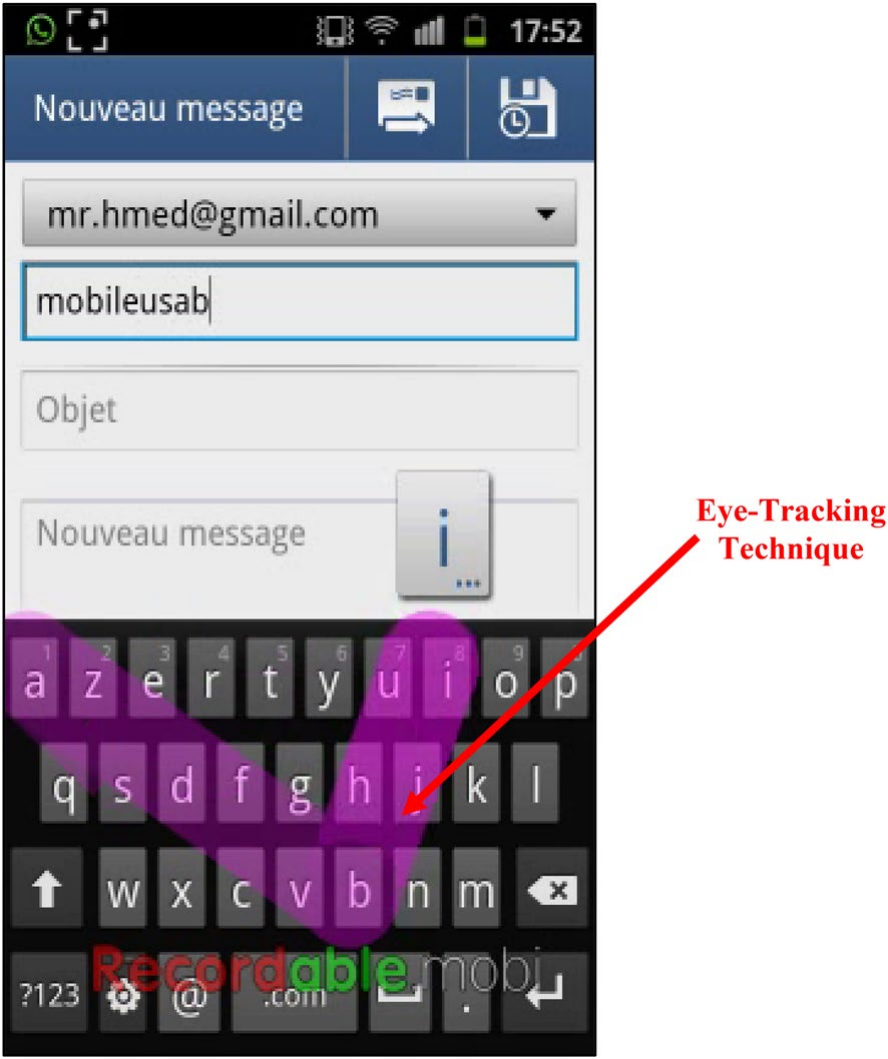
Sending an email on an Android device
- For Google Maps, the tasks include the navigation to a specific address from the position of each user, display the required time and distance by car, by bike, or on foot. Figures 7 and 8 show the launching of the Google Maps on Android and iOS devices.

- For Google Apps, three tasks have been created including: sending and receiving emails, the display and modification of an excel file in Google Docs, and the creation of an event on Google Calendar. Figures 5 and 6 show the execution of Google Apps tasks on Android and iOS devices.

**Fig. 7:**
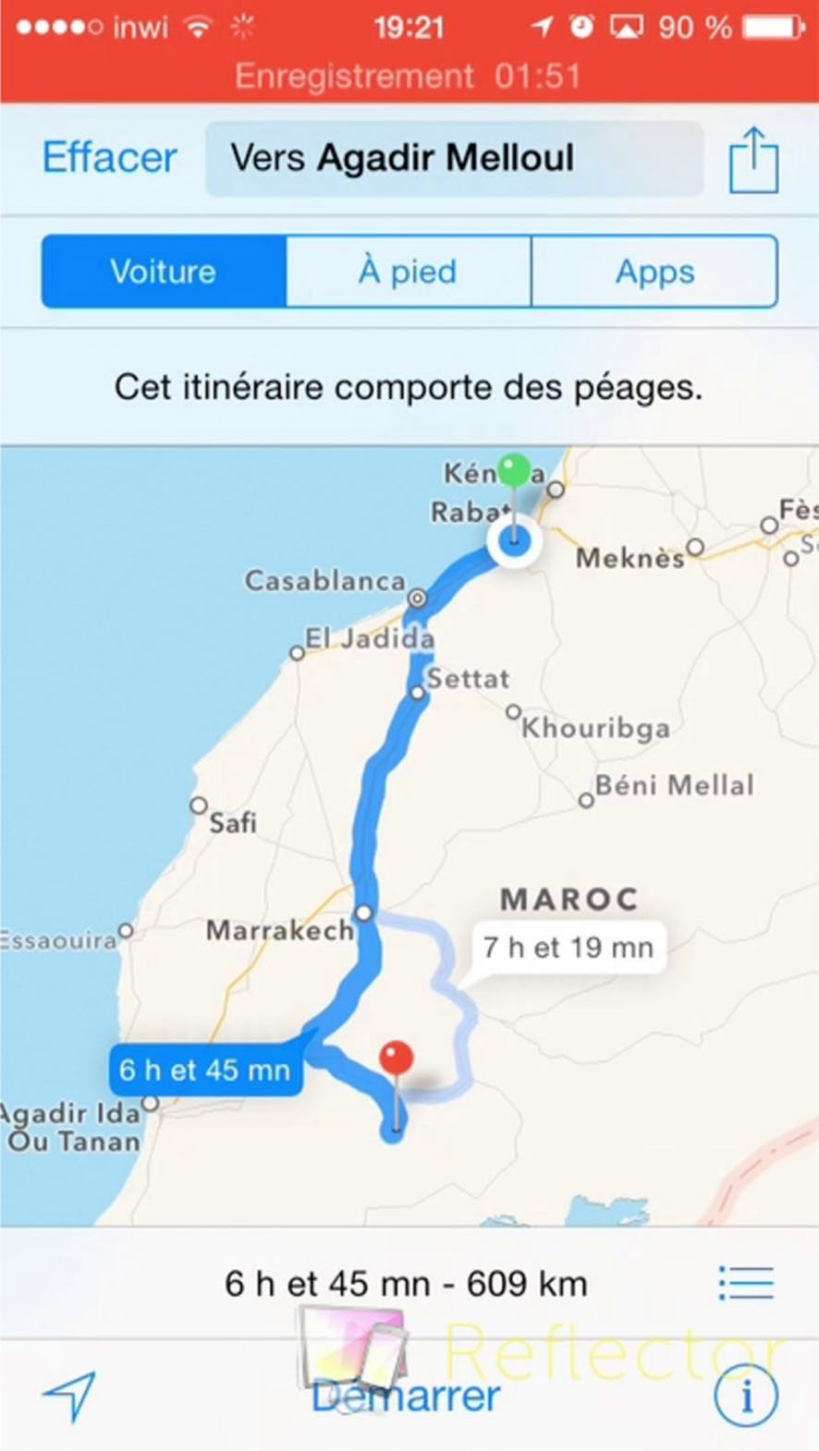
Google Maps on an iOS device

**Fig. 8:**
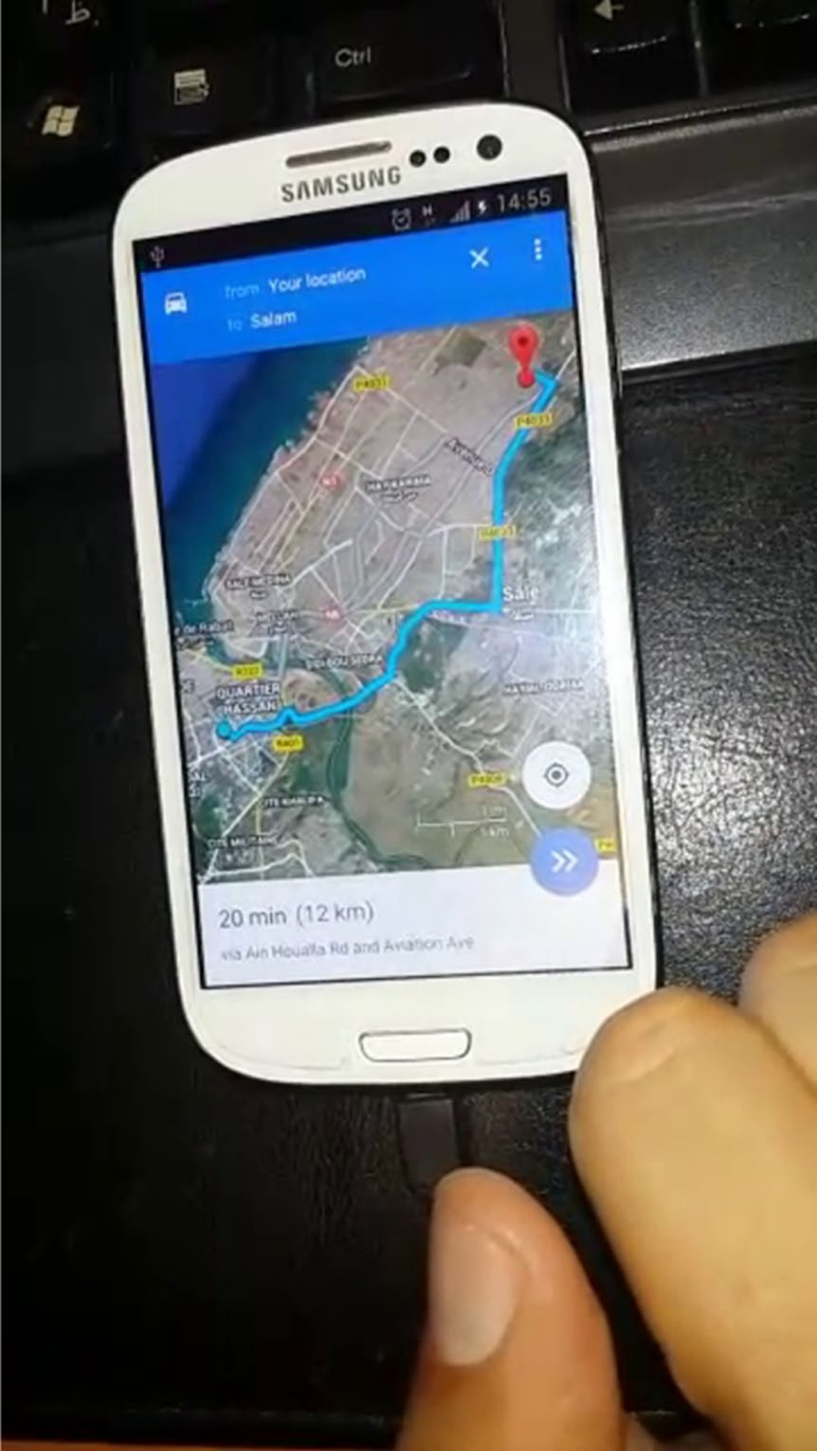
Google Maps on an Android device

Also as an instrument in this experiment, the use of two questionnaires: a General Information Questionnaire and the QUIS 7.0 for satisfaction measures, this last has a good recognition in the field of user satisfaction and is used in several mobile usability evaluation studies (Hussain and Kutar 2012a). The QUIS 7.0 designed by a group of researchers in Human–Computer Interaction at the University of Maryland was used to collect the user’s opinions and to evaluate their satisfaction on different aspects of an interface on a 9-point scale (Chin et al. 1988). It includes the following components: a demographic questionnaire, the evaluation of the system satisfaction via six scales, and the measures of nine specific interface factors: screen aspects, terminology and system information, learning aspects, system capabilities, technical user guides, online help and online tutorials, multimedia, conferencing, and methods of software installation. Each interface factor has a question as a major component followed by subcomponents questions, each of which rated on a scale of 1–9, with significant positive adjectives on the right, other negative on the left and NA (not applicable). The questionnaire also contains free text boxes for each section that allows the user to list his comments and provide feedbacks (Harper et al. 1990).

### Procedure

**Fig. 9:**
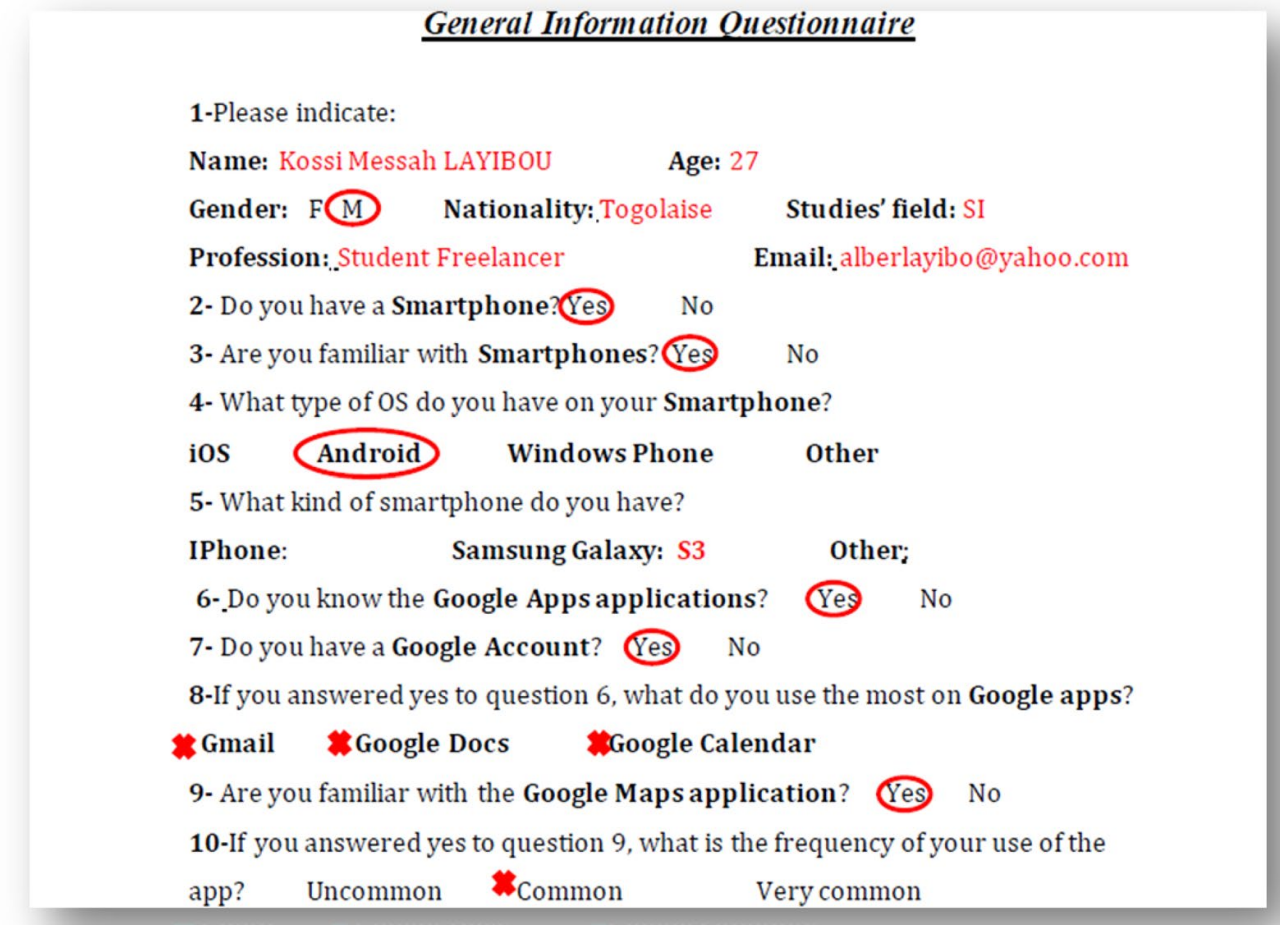
General information questionnaire

First, a General Information Questionnaire has been given to users in order to describe their knowledge of both apps and their familiarization with smart phones. Figure 9 show a screenshot of a part of this questionnaire. According to users’ answers, they had divided equally into two groups. Users, who have never worked with Google Apps but have already worked with Google Maps, were assigned to Google Maps group. This assignment is independent of the level of mastery of each application, in order to have a heterogeneous group of users: novice, experienced, and expert. The same is for people who have already worked with Google Apps; they were assigned to the Google Apps group.

After the assignment to groups, users started installing apps on their devices. All devices could connect to internet through a Wi-Fi with the exception of four users of both groups who had worked with 3G in order to see the influence of the variable bandwidth limitation on the usability. The participants had asked also to think aloud during the experiment via the recorders of their devices in addition to the eye-tracking technique that was used in order to see the movement of users’ eyes when performing tasks. We are interested in the way in which they think and interact with the apps. This had allowed each user to make his thoughts and ideas audible, which really helps to understand what is going on in his mind during the use of the apps. In addition, an expert user was selected on the basis of more than 1 year’s experience of using apps with the same device as in the experiment.

Different tools were used to record videos of the users’ tasks execution, such as: @screen for Android devices, Recordable.mobi for Eye-Tracking technique (Fig. 6), Reflector tool for iOS devices that was installed on Mac laptop as shown in Fig. 7.

**Fig. 10:**
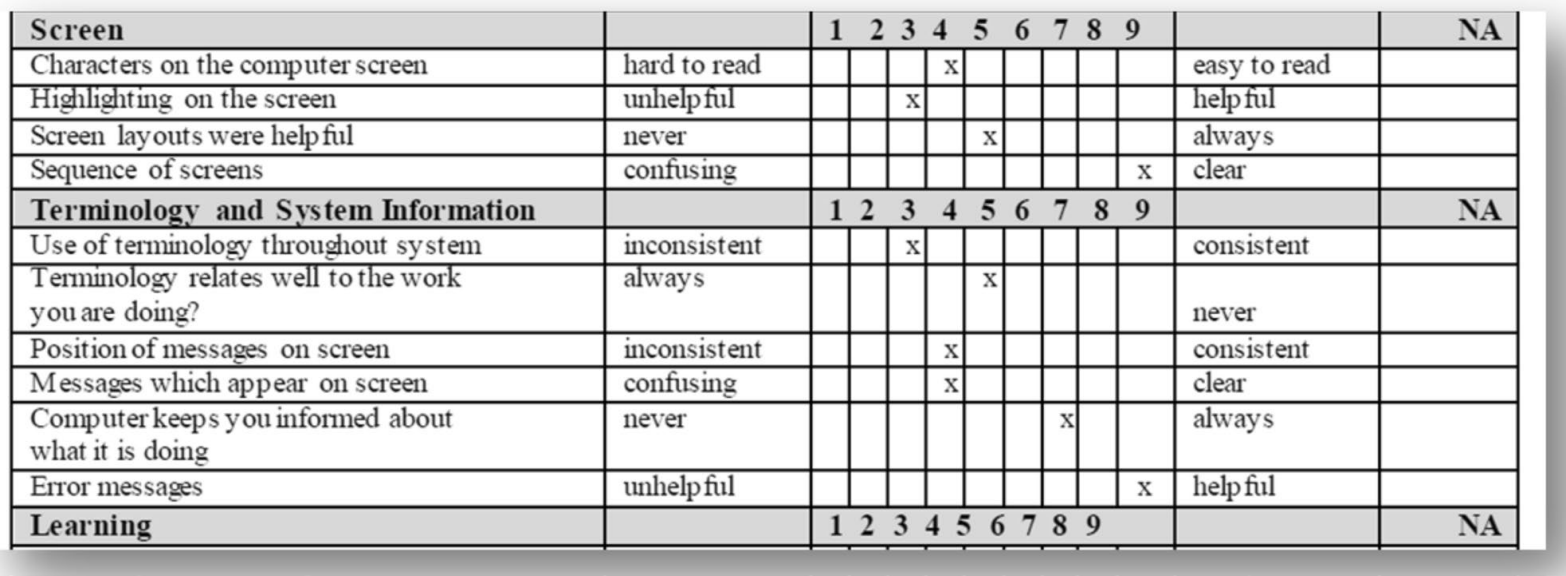
QUIS 7.0 questionnaire

Following the execution of tasks, users fill up the QUIS 7.0 as shown in Fig. 10. This experiment led to four deliverables for each participant: (1) recorded video, (2) recorded voice, (3) the QUIS 7.0 filled, and (4) the data collected. Note that we have taken the necessary permission from users to reproduce the experiment images and their pictures in this paper.

## Discussion and interpretation

This section discusses the results of the empirical validation of our framework, in particular those concerning the usability characteristic. The experiment report was based on the guidelines proposed by ISO 25062:2006 as a standard method for reporting results of usability evaluation.

Table 3 presents the objective measures (Table 2) derived from the video recordings of the execution of tasks. Table 4 presents the subjective user-satisfaction measures corresponding to the users’ answers to QUIS 7.0. The data analysis and graphs generation were conducted by means of the Statistical Package for the Social Sciences (SPSS) under a Microsoft environment. This discussion is structured to answer the two questions representing the aim of this experiment.

**Table 3:** Results of objective measurements of both applications Google Maps/Google Apps

Usability metric	Google Maps			Google Apps		
	Expert	Median	Std. deviation	Expert	Median	Std. deviation
Time to learn and use(s)	27	60	291.11	15	180	182.95
Time to install (s)	60	60	154.87	120	420	546.70
Response time (s)	14	14.00	49.08	70	60	199.93
Data entry time (s)	24	24	55.99	80	109	84.88
Number of errors	1	1	4.02	2	1	2.13
Tasks time (s)	126	180	480.04	150	540	585.89
Completion rate (%)	100	100	31.82	100	100	18.07
Number of voice support	0	0	0	0	0	0

**Table 4:** Results of subjective measurements of both applications Google Maps/Google Apps

Satisfaction measures	Google Maps			Google Apps		
	Mean	Median	Std. deviation	Mean	Median	Std. deviation
Overall reaction	5.64	5.41	2.15	6.35	7	1.67
Screen evaluation	5.87	6	1.94	7.03	7.42	1.43
Terminology and information	5.61	6.08	2.08	6.39	6.83	1.64
Learning	5.64	6.25	2.01	6.91	6.75	1.15
Application capabilities	5.89	6.12	2.05	5.72	6	1.28
Usability and UI	4.93	6.5	2.69	6.53	6.5	1.22
Technical manual and on-line help	4.5	4.5	2.42	6.27	6	2.12

- Question 1: What are the measures that have been impacted during the evaluation of the usability of mobile applications?

### Objective measures

Table 3 summarizes the median and the standard deviation of data collected for each measure and for the six predefined tasks of both apps as described in “Instrumentation” section. In addition, data relating to the expert were displayed in order to make the comparison. Comparisons are made relatively to the median because the mean is sensitive to extreme values unlike the median, which is less sensitive to extreme values.

According to Table 3, measures that have a large difference from the expert’s data, are *Time to learn and use* and *Tasks time* for Google Maps, in addition to *Time to install* and *Data entry time* concerning Google Apps. Such a large difference may be due to:

1. There were two novice users whose period of possession of their mobiles does not exceed 1 month,
2. there was also two users who do not have smart phones and worked on another ones, which required more *Time to learn and use,* and the same for *Data entry time,*
3. the two participants with Nokia smart phones who have found difficulties during the experiment for tasks relating to Google Maps. Therefore, they spent more than 7 min to search on Maps which explains the time taken to perform the tasks and the number of committed errors, and finally.
4. the four users who worked with 3G, from where the time spent in the installation and in the execution of tasks.

Also, concerning Google Apps, *Time to install* and *Tasks time* were respectively 120 as an expert value against 420 and 150 against 540; this was due to the fact that participants had to download and to install Google Calendar and Google Doc on their devices. In contrast with Google Maps, the participants had nothing to install except an update when it has been necessary.

**Fig. 11:**
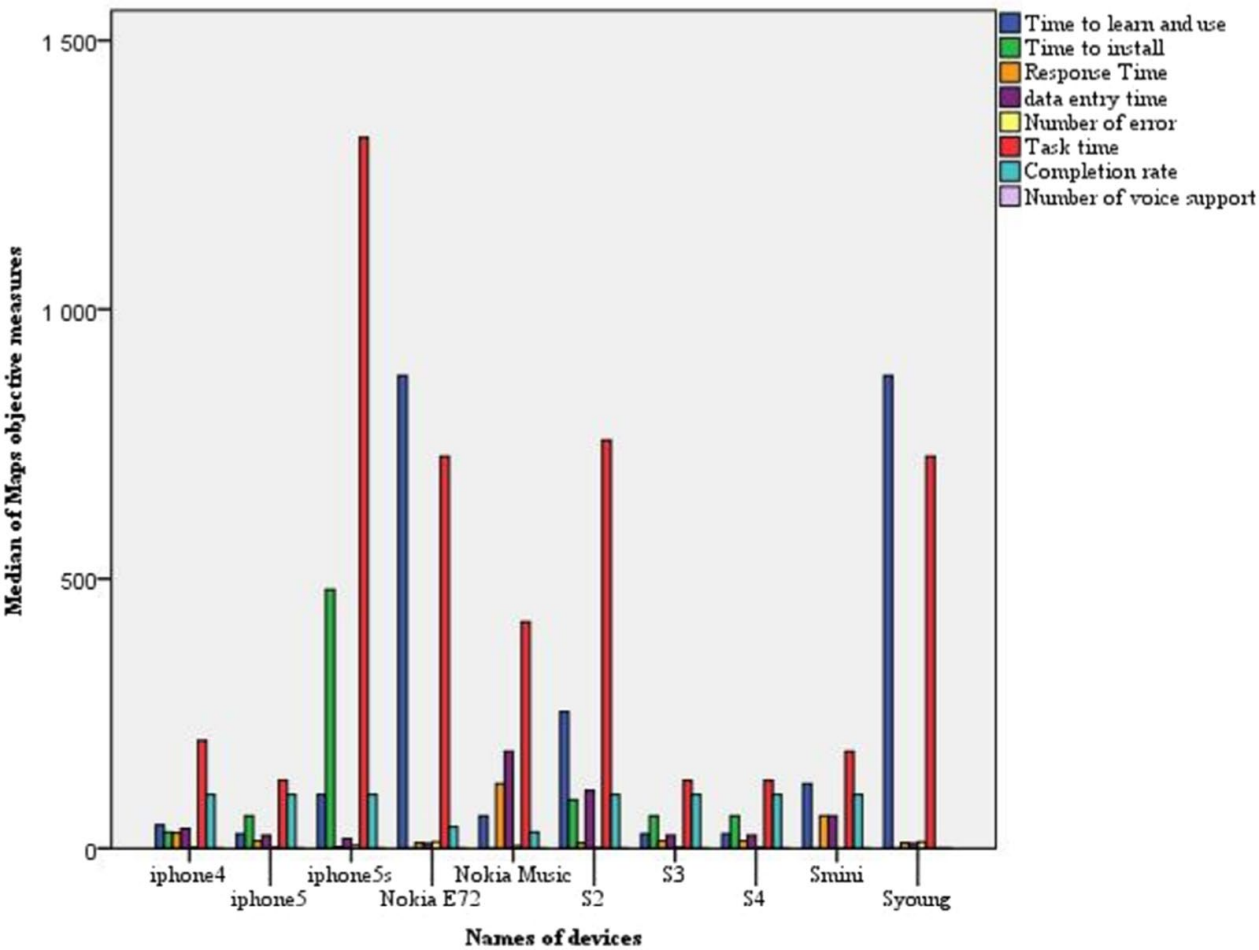
Median values of objective measures for Google Maps in terms of types of devices

**Fig. 12:**
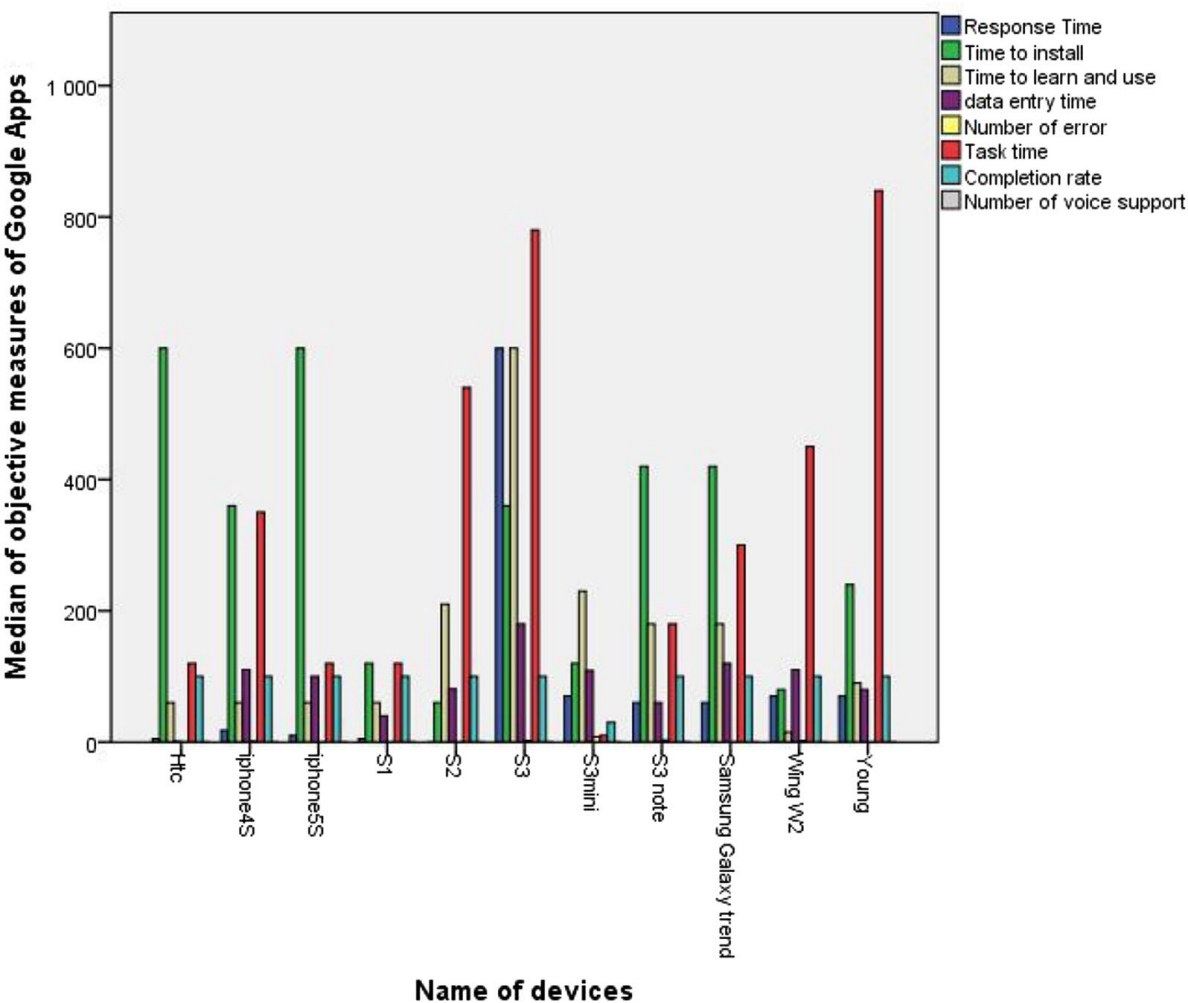
Median values of objective measures for Google Apps in terms of types of devices

Figures 11 and 12 show the relationship between the types of devices and the median of each objective measure for respectively Google Maps and Google Apps. According to Fig. 11, Google Maps was faster on iPhone 4/5, S3 and S4 than on Nokia E72, S young S2 and Nokia Music more particularly in terms of *Tasks time* and *Time to learn and use*. Figure 12 clearly shows that Google Apps in Htc, iPhone 5S, and S3 Note was faster and better than in S1, S2, S3 mini and S young more primarily in terms of *Time to install* and *Tasks time*. Therefore, the screen size, the display resolution, and the storage capacity of a mobile device have a very important role in the ease of use of a mobile application. In addition, smart phones need so much power for a good handling and quick response to user requests. For the *Completion rate* as shown in Table 3, the median was 100 for both Google Maps and Google Apps, which means that the majority of participants were able to complete their tasks and achieve goals, with the exception of:

- one person who could not install all the Google Apps applications because of the storage capacity of his device, and.
- those with Nokia who also did not complete the tasks of Google Maps under this type of device.

### Subjective user-satisfaction measures

This section concerns results obtained by analyzing the answers of participants to the QUIS 7.0 questionnaire. Table 4 shows the mean, the median and the standard deviation of subjective measures for both apps Google Apps and Google Maps. The questionnaire answers have been classified on a scale of 9: 9 means excellent, 6–8 means very good, 4–6 means good, 2–4 means fair and 1–2 means poor.

The overall reaction to Google Apps was very good (median and std deviation are equals to 7 and 1.67 respectively) and higher than those of Google Maps (median and std deviation are equals to 5.41 and 2.15 respectively) despite the ease and the simplicity of this application. It may be due to two reasons: (1) there were two novice users who were working with Google Maps for the first time; (2) there were two users with Nokia E72 devices, which are characterized by a very small screen and a limited storage capacity, which makes the use of the different tasks very difficult and not at all obvious.

The majority of participants were satisfied with Google Apps with a very good median value of 7 of the *overall reaction* measure. So, they found Google Apps very challenging and impressive especially on large screen size devices with high display resolution, a large memory and a good computing power such as S3, S4, S3Note, and iPhones. Except for one participant, he could not perform the tasks with his own device Wing W2 (system Android 4.2) because of the Android version that supports neither Google Calendar nor Google Doc, so he worked with another device. The same for Google Maps, the median was good with a value of 5.4; this is due to some participants who did not like the application on their smart phones because of the screen size and low memory as it is the case for S2, S3 mini, Nokia Music and Nokia E72.

**Fig. 13:**
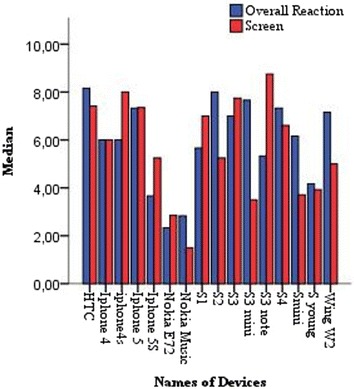
Median values of the screen evaluation and the overall reaction of the different types of devices

For the *Screen*, all participants gave at least 8 of 9 points to the *overall reaction* when they used S3, S4, S3 Note, iPhone 5, and iPhone 5 s which have large screens. However, as shown in Fig. 13, poor and fair values for *Screen evaluation* were given for Nokia E72, Nokia Music, S young, S3 mini, and S2 which screens are too small. Therefore, viewing and editing files for the case of Google Apps were stressful and difficult as you have to go in rotation mode of the screen for a better visibility and a good display. The disabled participant was satisfied with S3 Note as it has a large screen that allowed him to work easily and quickly.

Regarding *Learning and technical On-line help*, they had respectively median values of 6.25 and 4.5 for Google Maps against 6.75 and 6 for Google Maps. This may be explained by the non-presence of help messages and online user manuals of the apps for certain type of devices, particularly for Google Maps under Nokia devices where users had to fumble for how to accomplish the various tasks. However, these two measures have a major role for easy and optimal use in terms of time, since they correspond to learn how to operate the app with help messages on the screen and online user guides. For example, Google Apps was impossible to use under Nokia E72 devices because of the screen size and low memory. For Google Maps, it was very difficult to use it under this type of devices. Therefore, the novice users were blocked at the beginning and they took the needed time looking how to operate this app and to become familiar with it on their devices.

Figure 13 shows that participants were satisfied and were very pleased with smart phones that are quick with a large screen size and a very high resolution as S3, S4, S3 Note, iPhone 5/5 s, unlike the other types of devices that are very slow in addition to the screen size that was stressful with a poor quality information display.

Whatever the simplicity of a mobile application or the work to be done on a smartphone, all depends primarily of its characteristics. So, despite the complexity of Google Apps tasks compared to those of Google Maps which have been very easy, most users have enjoyed working with Google Apps seen the power of their devices, their speed, and also the high-quality screen resolution.

- Question 2: What are the other limitations that influence the usability of mobile applications compared to limited user interface?

In summary, the objective measures identified by comparing to the expert data of both apps are: *Time to install*, *Time to learn and use*, *Tasks time* and *Data entry time*. Concerning the subjective measures we focused on the *Overall Reaction*, the *Screen Evaluation* and the *Technical Online Help* according to the median value. Therefore, all these measures depend on several factors as indicated in “Using ISO 9126 for software quality in mobile environments” section: the mobile context, the connectivity, the data entry methods, the presence of technical online help, the characteristics of devices especially the screen size, the display resolution, low memory, type of keyboard, etc.

According to the mobile users involved in this experiment, the screen size, the display resolution and the storage capacity were the main limitations of mobile devices that affect the usability of apps. Since, the higher the screen resolution, the more the user has space for playing games, reading text, viewing files and reports, taking pictures and recording videos. In addition, the low storage capacity of mobile devices affects also the ease of use of apps. This problem becomes a handicap especially for smart phones that do not have a memory card, which blocks the user and does not let him install some large size apps. However, the influence of the other mobile limitations such as the connectivity, the mobile context and the data entry methods on the usability of apps must be taken into account during the usability evaluation.

As a conclusion of this experiment, the obtained results confirm what we have found during the application of the framework on usability external metrics: the limited user interface has a strong influence on the majority of the external metrics of the usability characteristic (21 metrics of 27 metrics are influenced). Regarding limited storage capacity, it has a weak influence (2 metrics of 27 metrics) on the usability characteristic when compared to other ISO 9126 quality characteristics (ISO 9126-2 2001). These results are considered normal since usability is related to the use challenges of the software product, and these challenges depend on the difficulties encountered when the user interface is exploited.

In addition to the two hardware limitations, other issues related to the software and to the mobile context are presented: the absence of technical manuals and online help, the limited and variable bandwidth of networks in addition to complicated data entry methods. Therefore, mobile application designers and developers must make available to users easy and simple interfaces that can be used by any kind of users: novice, experienced and experts. These applications must also be equipped with user guides and online manuals.

Therefore, the evaluators of usability characteristic should take these mobile limitations into consideration by using ISO 9126 measures or by suggesting new measures for the mobile environments.

### Validity evaluation

The empirical validation we have performed is limited by a number of factors (Johnson 1998b; Petrie et al. 1998). Threats related to the construct, to the internal and to the external validity, are below.

#### Threats to construct validity

“The construct validity is a matter of judging if the treatment reflects the cause construct and the outcome provides a true picture of the effect” (Dumas and Redish 1999; Nielsen and Landauer 1993). Since the objective measures are collected through video recordings and subjective measures via questionnaires, here the data collected reflects the reality.

#### Threats to internal validity

Internal validity is related to the validity of the study within the used environment and the reliability of obtained results. The Empirical Evaluation had performed in a controlled environment, which constitutes a threat to internal validity because users were far from any interruptions, noise. However, users have taken a little time to become familiar with the environment before starting the experiment; as a result, this threat could be minimized.

#### Threats to external validity

The external validity is related to the generalization of findings. The sample of the experiment was small (32 participants) which constitute a clear threat to external validity. Another limitation of this experiment is that it was based on two widely used mobile applications: Google Apps and Google Maps that were proposed by participants. Therefore, it is difficult to generalize the results for other mobile apps and mobile sites.

## Conclusion and future work

This paper has presented an empirical evaluation of our framework developed on the use of the software quality standard ISO 9126 in mobile environments, especially the effects of mobile limitations (limited user interface, frequent disconnection, lower bandwidth, etc.) on the usability of apps by means of ISO 25062 and ISO 9241 standards. To do this, an experiment has been conducted by giving 32 users a set of tasks to be performed on their devices and allowing them to think aloud while using both Google Maps and Google Apps. The aim was to identify and to highlight the usability issues when using apps. In this experiment, we have collected objective measures using a set of measures and by video recordings, as well as subjective measures via the QUIS 7.0 questionnaire. The results obtained were analyzed and interpreted on the basis of the expert data, the user’s descriptions, and devices’ characteristics. Hence, we identified a set of challenges when using apps related to the characteristics of the device (Hardware) such as the screen size, the display resolution and the capacity of memory which validate the findings of our framework. In addition, other issues were identified which are related to the application itself (Software) as the presence of online help and user guides, the use of simple data entry methods, etc. Thus, owning a smart phone with a large screen is a good thing because this screen is very convenient and may make everything easier to use. It may serve as an e-book reader, and it may be turned into a console to play games easily. In addition, the problems related to the software, as user guides and online help, must be made available especially to novice users in order to learn how to operate most apps.

 Further research works will be initiated to carry out this experiment in the field on the basis of a large sample of users, for detection of new usability issues of apps. Thereafter, empirical evaluation should be conducted to validate the other analytical findings, concerning the reliability, efficiency, and functionality characteristics.
